# Impact of an innovative tuberculosis financing and payment model on health service utilization by tuberculosis patients in China: do the poor fare better than the rich?

**DOI:** 10.1186/s40249-019-0559-z

**Published:** 2019-06-11

**Authors:** Di Dong, Wei-Xi Jiang, Qian Long, Fei Huang, Hui Zhang, Jia-Ying Chen, Li Xiang, Qiang Li, Sheng-Lan Tang, Henry Lucas

**Affiliations:** 1 Global Health Research Center, Duke Kunshan University, Kunshan, Jiangsu China; 2 National Center for Tuberculosis Control and Prevention, China CDC, Beijing, China; 3 School of Policy and Management, Nanjing Medical University, Nanjing, Jiangsu China; 4 Tongji Medical College of Huazhong University of Science and Technology, Wuhan, Hubei China; 5 School of Public Health, Xi’an Jiaotong University, Xi’an, Shaanxi China; 6 Duke Global Health Institute, Duke University, Durham, NC USA; 7 Institute of Development Studies, University of Sussex, Brighton, UK

**Keywords:** Health service use, Tuberculosis, Financing and payment model, Case-based payment

## Abstract

**Background:**

Tuberculosis (TB) prevalence is closely associated with poverty in China, and poor patients face more barriers to treatment. Using an insurance-based approach, the China-Gates TB program Phase II was implemented between 2012 and 2014 in three cities in China to improve access to TB care and reduce the financial burden on patients, particularly among the poor. This study aims to assess the program effects on service use, and its equity impact across different income groups.

**Methods:**

Data from 788 and 775 patients at baseline and final evaluation were available for analysis respectively. Inpatient and outpatient service utilization, treatment adherence, and patient satisfaction were assessed before and after the program, across different income groups (extreme poverty, moderate poverty and non-poverty), and in various program cities, using descriptive statistics and multi-variate regression models. Key stakeholder interviews were conducted to qualitatively evaluate program implementation and impacts.

**Results:**

After program implementation, the hospital admission rate increased more for the extreme poverty group (48.5 to 70.7%) and moderate poverty group (45.0 to 68.1%), compared to the non-poverty group (52.9 to 64.3%). The largest increase in the number of outpatient visits was also for the extreme poverty group (4.6 to 5.7). The proportion of patients with good medication adherence increased by 15 percentage points in the extreme poverty group and by ten percentage points in the other groups. Satisfaction rates were high in all groups. Qualitative feedback from stakeholders also suggested that increased reimbursement rates, easier reimbursement procedures, and allowance improved patients’ service utilization. Implementation of case-based payment made service provision more compliant to clinical pathways.

**Conclusion:**

Patients in extreme or moderate poverty benefited more from the program compared to a non-poverty group, indicating improved equity in TB service access. The pro-poor design of the program provides important lessons to other TB programs in China and other countries to better address TB care for the poor.

**Electronic supplementary material:**

The online version of this article (10.1186/s40249-019-0559-z) contains supplementary material, which is available to authorized users.

## Multilingual abstracts

Please see Additional file 1 for translations of the abstract into the five official working languages of the United Nations.

## Background

Tuberculosis (TB) prevalence is closely associated with poverty in China. The 2010 National Tuberculosis Prevalence Survey shows that TB prevalence is much higher in rural areas than urban areas (163 vs 73 per 100 000), and higher in the less developed western region than the developed eastern region (212 vs 66 per 100 000) [1]. Some 83% of TB patients live in households with incomes below the regional median, and the average per capita household income of rural TB patients is 50% less than the local regional median [2].

Low-income TB patients in China face greater financial barriers to quality treatment and have lower treatment adherence [3, 4]. Currently, TB treatment guidelines by the World Health Organization (WHO) and Chinese Center for Disease Control and Prevention (China CDC) recommend that rifampicin-sensitive newly diagnosed TB patients should receive 6 months of outpatient treatment and relapse TB patients 8 months [5–7]. A national survey in 2010 indicated that 10% of TB patients had intermittent treatment, and another 22% terminated before completing treatment. 15% of patients who terminated treatment reported that financial difficulties were the cause for their poor adherence [1]. TB patients with lower household incomes and education levels were more likely to report non-compliance [3]**.** Since 1990s, first-line anti-TB drugs and basic diagnostic tests during the standard treatment course are provided free of charge [8]. However, the overall cost of TB treatment is substantial, and poor TB patients are more likely to have catastrophic health expenditure. A study in three cities in China found that over 94% of households in the poorest quintile, compared to 43% in the richest quintile, had catastrophic expenditure due to TB treatment [4].

Several factors add to the financial barriers for poor patients to access TB treatment. First, many poor patients are covered by the New Rural Cooperative Medical Scheme (NCMS) or Urban Resident Basic Medical Insurance (URBMI), which have less comprehensive coverage and lower reimbursement rates for TB services compared to the Urban Employee Basic Medical Insurance (UEBMI) [9]. Second, low-income rural TB patients more often go to smaller regional hospitals that have a lower capacity to diagnose and treat complex cases. This may lead to over-provision of unnecessary and often expensive services not covered by the government free treatment policy or health insurance, such as computed tomography (CT) scans, branded second-line anti-TB drugs, liver protection and other ancillary drugs, the cost of which may further deter poor patients from completing treatment [10]. In addition, many poor patients live in rural and remote areas. Seeking treatment may therefore require travelling a considerable distance, incurring substantial transportation and accommodation costs [10] and possible loss of income.

To improve access to TB care and reduce the financial burden, particularly in rural areas, the China-Gates TB program Phase II was implemented between 2012 and 2014 in three prefectures from eastern (Zhenjiang), central (Yichang) and western (Hanzhong) China. The program context and intervention details have been documented elsewhere [8]. Briefly, the new TB financing and payment model includes: 1) increasing health insurance reimburse rates for hospitalization and outpatient TB services to 70%; 2) changing the provider payment method to case-based payment from the current fee-for-service, to incentivize cost containment by TB-designated hospitals. For practical reasons, the case-based payment design included distinct payment packages for inpatient and outpatient services; 3) providing transportation and subsistence allowances to TB patients who adhered to treatment (including those without health insurance). Equity assessment at study baseline revealed that rural residents in project counties had less service utilization, but more out-of-pocket payment per hospital admission compared to urban employees and urban residents [11]. Analysis of the rural TB patients enrolled in the NCMS also revealed low reimbursement rate and high financial risk [12]. Service access and financial risk protection for TB patients with lower socioeconomic status were identified as major gaps in TB care [8].

There is rich international literature suggesting that public subsidies for health programs frequently benefit richer more than poorer people [13, 14]. Whether a health insurance-based approach can effectively target the poor and improve equity is therefore of great concern. This study aims to address this issue by considering the impact of the China-Gates TB program (Phase II) on service utilization, treatment adherence and patient satisfaction across three income groups identified as consisting of those living in: extreme poverty, moderate poverty and non-poverty.

## Methods

### Study setting

Quantitative data were obtained from cross-sectional surveys with TB patients at the baseline of the program in 2012 and final evaluation in 2014. Three counties (one low-income, one middle-income and one high-income) were selected in each of the three project prefectures (Zhenjiang, Yichang and Hanzhong). In each of the above 9 counties, TB patients who had been diagnosed for more than 6 months (8 months for relapse patients) were identified from the China CDC‘s TB information management system (TBIMS), which is a national registry for the compulsory reporting and management of confirmed TB cases. Ninety TB patients were randomly sampled from each of the nine counties’ TBIMS. When less than 90 patients were present in a county, all patients were sampled. One of the county was excluded from analysis due to an unexpected restructuring of regional TB management system, leading to incomparable data at baseline and final evaluation. Face-to-face interviews were conducted using a structured questionnaire, which included information on patient socio-economic background, treatment history, and satisfaction. A total of 788 patient questionnaires were analysed from the baseline survey and 775 from the final evaluation survey.

### Income grouping and poverty status

The self-reported per capita annual income of a patient’s household was used for income grouping. Following the World Bank’s definition of extreme poverty globally and the poverty line for upper-middle income countries, those with incomes less than USD 1.9 a day (RMB 4369 per annum) were classified as being in extreme poverty, those with incomes between USD 1.9 to 5.5 a day (RMB 12647 per annual) were classified as being in moderate poverty, and the rest were classified as non-poverty households [15]. This classification was validated to be relevant to local poverty standards in Zhenjiang, Yichang and Hanzhong. The local living assistance standards for urban residents announced by the Department of Civil Affairs all three cities in 2014 were similar to or higher than the World Bank standard of extreme poverty.

### Measuring service utilization, adherence, and patient’s satisfaction

Inpatient and outpatient service utilization indicators were analysed, including the hospital admission rate, average number of hospital admissions per patient, rate of hospital re-admission within 3 days of discharge, average length of stay for the first admission, and average number of outpatient visits during a treatment course (6 months for new patients, and 8 months for relapse patients). Medication adherence was characterized using the following indicators: proportion of patients refusing treatment, taking medications as prescribed, and terminating treatment. Patient satisfaction was measured relating to: treatment outcome, reimbursement rate, reimbursement procedures, attitudes of doctors and nurses.

### Statistical analysis

Utilization of inpatient and outpatient services, patient adherence and satisfaction were examined for each poverty status at baseline and at final evaluation using descriptive statistics.

The effect of the program on the rate of hospital admission, rate of re-admission within 3 days after discharge, and proportion of patients with good medication adherence were analysed using multivariate logistic regression model. Number of outpatient visits over the whole treatment course were analysed using Poisson regression. All regression models included the following explanatory variables: period (baseline, final evaluation), poverty status and the interaction term between poverty status and period as explanatory variables. All models were controlled for county fixed effects and cluster-robust standard errors were used for statistical testing. A set of control variables were included in all regression models, including gender, age, marital status, TB type (new, relapse), education level, employment status and health insurance type. The models estimated were thus of the form:

Outcome = *f* (poverty status, period, poverty status*period, control variables, county fixed effect).

All analyses were performed using Stata 14 (Version 14, StataCorp, College Station, TX. Statistical significance were assessed at *P* = 0.05.

### Qualitative interviews and analyses

Qualitative data were obtained during the final evaluation period of the program in 2014. Semi-structured in-depth interviews were conducted with city- and county-level health administrators (*n* = 12), health insurance managers (*n* = 20) and hospital managers (*n* = 12) to understand their perceptions of the program impacts on poor TB patients’ service utilization and equity. Two focus group discussions (FGDs) were held with healthcare providers in TB designated hospital and primary care doctors in each study county to explore their views of the program impacts on service provision and patient adherence. Each group consisted of 5–6 physicians and nurses who provided outpatient and/or inpatient TB care and were responsible for TB patient management. In addition, two FGDs with TB patients were organized in each study county to gain understandings of patients’ care seeking and treatment experiences and level of satisfaction. TB patients were quota sampled based on their gender, household income and type of health insurance coverage. Each group consisted of 6 TB patients and was held in a private room in the hospital. All interviews were conducted by experienced evaluation team members and recorded after obtaining the permission of participants.

The Framework approach [16] was used to analyse the qualitative data. A framework constructed using the topic guide, field notes and categories emerging from the transcripts, was applied to the data to identify themes. Data from different stakeholders and other sources were triangulated. The key findings were also validated by external consultation.

## Results

A total of 788 patient questionnaires were analysed from the baseline survey and 775 from the final evaluation survey. The socio-economic characteristics of patients were similar in both samples, except for their health insurance coverage (Table 1). At baseline, 34% of patients were classified as in extreme poverty and 42% in moderate poverty compared to 36 and 40% at final evaluation.

**Table 1:** Characteristics of TB patients at baseline and final evaluation

Sample Characteristics	Baseline (*n* = 788)	Final evaluation (*n* = 775)	*P* value
Gender (%)			
Male	74.8	70.7	0.070
Age (%)			
< 30	6.2	7.4	0.070
30–59	46.7	41.0	
≥ 60	47.1	51.6	
Marriage (%)			
Married	80.3	78.2	0.290
Residence (%)			
Rural	92.5	90.0	0.083
Patient category (%)			
New patient	81.4	82.2	0.670
Education level (%)			
None	20.2	25.0	0.052
Primary	32.9	33.6	
Secondary	34.0	28.7	
High school and above	12.9	12.8	
Insurance type (%)			
UEBMI	6.4	9.3	0.000
URBMI	2.8	3.2	
NCMS	87.8	82.2	
URRBMI	0.0	2.5	
Private insurance	1.8	0.4	
other insurance	0.3	0.3	
no insurance	1.0	2.2	
Employment (%)			
Currently working (including farming)	53.3	49.0	0.092
Income level (%)			
Extreme poverty	34.0	36.1	0.642
Moderate poverty	42.0	40.0	
Non-poverty	24.0	23.9	

*UEBMI* Urban employee basic medical insurance, *URBMI* Urban resident basic medical insurance, *NCMS* New corporative medical scheme, *URRBMI* Urban rural resident basic medical insurance

After program implementation, utilization of inpatient and outpatient services increased, but to different extents in different poverty groups. The hospital admission rate increased from 48.1 to 68.1% among all patients, and the increase was greater for the extreme poverty and moderate poverty groups (Table 2). The average number of hospital admissions decreased from 1.4 to 1.2, and the rate of re-admissions decreased from 23.0 to 15.5%. The length of stay for the first hospital admission increased in the extreme and moderate poverty groups, but decreased in the non-poverty group. The number of outpatient visits increased from 4.8 to 5.7 among all patients, and the increase was highest in the extreme poverty group.

**Table 2:** Inpatient and outpatient service utilization at baseline and final evaluation (by income group)

Indicator	Period	Extreme poverty	Moderate poverty	Non-poverty	Total
Hospital admission rate (%)	Baseline	48.5	45.0	52.9	48.1
	Final evaluation	70.7	68.1	64.3	68.1
Average number of hospital admissions	Baseline	1.5	1.3	1.3	1.4
	Final evaluation	1.3	1.2	1.2	1.2
Average rate of hospital re-admissions (%)	Baseline	29.2	17.5	23.0	23.0
	Final evaluation	21.2	11.9	12.6	15.5
Average length of stay of the first admission (days)	Baseline	21.1	22.6	26.1	23.0
	Final evaluation	24.0	24.7	25.6	24.7
Average number of outpatient visits during treatment course^a^	Baseline	4.6	5.0	4.8	4.8
	Final evaluation	5.7	5.7	5.8	5.7

^a^Only patients who had been diagnosed as TB for more than 6/8 months at the time of survey were included for analysis of outpatient visits

After program implementation, medication adherence improved the most in the extreme poverty group (Table 3). The proportion of patients refusing treatment decreased in the extreme poverty and non-poverty groups, but not in the moderate poverty group. The proportion of patients taking medication on schedule as prescribed increased by 15 percentage points in the extreme poverty group, and 10 percentage points in the other two groups. The proportion terminating treatment also decreased the most in the extreme poverty group, followed by the moderate poverty group.

**Table 3:** Program effect on patients’ treatment adherence and satisfaction

Indicator	Period	Extreme poverty	Moderate poverty	Non-poverty	Total
Proportion of patients who refused treatment (%)	Baseline	4.9	2.4	2.7	3.3
	Final evaluation	2.9	2.9	1.6	2.6
Proportion of patients taking medications as prescribed (%)	Baseline	75.4	81.6	81.0	79.3
	Final evaluation	90.5	93.1	92.3	92.0
Proportion of patients terminating treatment (%)	Baseline	19.0	13.0	15.9	15.7
	Final evaluation	5.9	4.9	6.0	5.5
Proportion of patients who satisfy with the treatment outcome (%)	Baseline	94.7	92.1	93.1	93.2
	Final evaluation	96.8	96.1	98.4	96.9
Proportion of patients who satisfy with the reimbursement rate (%)	Baseline	86.2	86.9	88.3	87.0
	Final evaluation	90.9	89.5	89.0	89.9
Proportion of patients who satisfy with the reimbursement process (%)	Baseline	93.1	90.5	92.3	91.8
	Final evaluation	93.3	90.6	93.3	92.3
Proportion of patients who satisfy with doctor’s attitude (%)	Baseline	96.1	96.6	99.0	97.1
	Final evaluation	99.1	98.2	99.2	98.8
Proportion of patients who satisfy with nurse’s attitude (%)	Baseline	96.9	97.3	99.0	97.6
	Final evaluation	99.1	97.8	98.4	98.4

Over 90% of patients reported satisfaction with treatment outcome, procedure, and the attitudes of doctors and nurses in both baseline and final evaluation surveys, and the proportion satisfied were slightly higher at final evaluation compared to baseline (Table 3). Reimbursement rates were satisfactory to the smallest proportion of patients but were still seen as acceptable to around 87% before and 90% after program implementation. The satisfaction did not seem to vary by poverty group.

The multivariate regression results are shown in Table 4. As expected, the overall hospitalization rate was substantially higher for members of the non-poverty group (*OR* = 1.44, *P* = 0.05). However, while program implementation appears to have considerably increased the hospitalization rate for all income groups (*OR* = 2.83, *P* = 0.01), the increase was much lower for the non-poverty group (*OR* = 0.56, *P* = 0.04), indicating increased equity of access. The re-admission rate did not seem to change after the program and there were no significant variations across poverty groups. The number of outpatient visits increased for all groups, and intra-group differences were not significant. The proportion of patients with good medication adherence improved significantly (*OR* = 2.88, *P* = 0.01), and there were again no significant differences across poverty groups. Patient satisfaction with treatment outcomes improved after the program but satisfaction with the reimbursement rate did not change.

**Table 4:** Program effect for different income groups

	Hospital admission		Rate of re-admission		Average number of outpatient visits		Good medication adherence		Satisfaction with treatment outcomes	
	Odds ratio	*P* value	Odds ratio	*P* value	Coefficient	*P* value	Odds ratio	*P* value	Odds ratio	*P* value
Poverty status (Moderate poverty as reference group)										
Extreme poverty	1.1	0.7	2.2	0.06	−0.1	0.21	0.7	0.23	1.5	0.07
Non-poverty	1.4	0.05	1.5	0.08	−0.1	0.27	0.9	0.55	1.3	0.48
Period (baseline as reference group)										
Final evaluation	2.8	0.01	0.6	0.11	0.1	0.01	2.9	0.01	2.0	0.04
Income level* Period										
Extreme poverty *Final evaluation	1.0	0.98	1.1	0.9	0.1	0.16	1.1	0.88	0.7	0.44
Non-poverty *Final evaluation	0.6	0.01	0.8	0.46	0.1	0.16	0.9	0.72	2.6	0.16
Gender (male as reference group)										
Female	0.7	0.01	1.2	0.46	0.0	0.28	1.1	0.62	1.5	0.16
Age group (< 30 as reference group)										
30–59	0.9	0.82	2.7	0.01	0.0	0.46	0.9	0.68	0.8	0.58
60+	1.2	0.08	1.0	0.83	0.0	0.89	1.0	0.83	1.9	0.08
Type of TB (newly diagnosed as reference group)										
Relapse	1.0	0.76	1.2	0.49	−0.1	0.14	0.6	0.01	0.5	0
Marital status (single as reference group)										
Married	1.0	0.89	1.2	0.52	0.1	0.23	1.1	0.56	0.8	0.57
Education (no formal education as reference group)										
Primary	1.0	0.68	0.9	0.68	0.0	0.30	1.0	0.97	1.0	0.95
Secondary	1.1	0.67	1.2	0.46	0.1	0.21	1.2	0.52	1.1	0.87
≥ High school	1.0	0.84	1.2	0.64	0.1	0.16	2.1	0.01	0.5	0.04
Insurance type										
UEBMI	0.4	0.04	1.0	0.96	0.0	0.86	1.6	0.17	0.5	0.35
NCMS	0.4	0.01	1.3	0.46	0.0	0.64	1.4	0.38	0.8	0.82
URRBMI	0.3	0	1.4	0.65	0.1	0.06	1.9	0.59		
Private	1.1	0.91			0.0	0.84	0.7	0.52		
No insurance	0.4	0.06	0.3	0.07	−0.1	0.70	1.3	0.46	0.8	0.85
Other insurance	0.9	0.96	1.8	0.69	0.4	0.01	0.6	0.59		
Currently working (currently no working as reference group)	0.6	0	0.9	0.57	0.0	0.95	0.8	0.33	1.3	0.38

*UEBMI* Urban employee basic medical insurance, *URBMI* Urban resident basic medical insurance, *NCMS* New corporative medical scheme, *URRBMI* Urban rural resident basic medical insurance

The qualitative results support the quantitative findings on increased outpatient and inpatient TB service use, and allow identification of some of the underlying reasons. First, most health administrators, health insurance managers, hospital managers and TB care providers thought that the increased health insurance reimbursement rate for TB care and the simplified reimbursement procedure for patients had a positive impact on service use, particularly among poor TB patients. Several TB care providers explained that the reimbursement rate for TB outpatient care was low before the introduction of the program, for example, only 20% of eligible expenditure would be reimbursed by NCMS, as compared to 80% after program implementation. Most TB patients also expressed their satisfaction with the increased reimbursement rate. Second, hospital managers mentioned that an upgraded IT system for case-based payment allowed patients to receive insurance reimbursement at the time of bill payment. Previously, paying a substantial deposit at admission or paying out-of-pocket while receiving treatment placed a major burden on poor patients. Some terminated treatment due to an inability to afford such payments even though a large proportion was expected to be reimbursed subsequently by insurance. Third, patients found transport and subsistence allowances provided effective motivation to undertake follow-up visits, especially for poor patients living in remote rural or mountainous areas. Travelling to a hospital could take many hours and entail significant costs, sometimes requiring an over-night stay. The allowances partially offset such costs, and disbursement of a lump sum payment upon completion incentivized treatment adherence.

*The current direct exemption of reimbursable expenses (at time of bill payment upon discharge) is good for poor patient, and rich patients may not care*. (TB hospital manager)

*I received RMB 180 for transportation and nutrition allowance. It helps me. Maybe you (policy maker) can consider giving more for those who live far away.* (TB patient, FGD)

Some hospital managers and providers considered the design of the health insurance reimbursement package to be in line with the standard TB clinical treatment pathway, which to some extent improved quality of TB care and case management. Several providers said that after program implementation, they prescribed TB treatment-related tests according to the clinical guideline and strengthened coordination with primary healthcare providers to follow up TB patients and encourage them to attend scheduled hospital visits. In FGDs with TB patients, many patients said they had frequent contacts with healthcare providers.

*Before the program, we only did some of the tests (like liver function test, blood test, kidney function) occasionally. After patients are discharged, we didn’t follow them anymore. Now, there is emphasis on standard treatment and quality, so we have improved on tests. Those necessary tests are done every month. If patients have adverse reactions, they can receive timely treatment.* (TB doctor, FGD)

*Now after a patient leave the hospital, we inform the village doctor to supervise the patient, and call the patient every month to remind him/her of the follow-up visit.* (TB doctor, FGD)

## Discussion

Overall, the China-Gates TB program Phase II has improved inpatient and outpatient TB care access, reduced re-admissions, enhanced medication adherence, and improved patient’s satisfaction with treatment outcomes. It was also found to be pro-poor and to have improved equity in inpatient TB care access across different poverty groups: the program effects on hospital admissions were greater for the extreme poverty and moderate poverty groups, compared to the non-poor group. Program effects on outpatient service utilization were similar across different groups, and number of outpatient visit did not correlate with poverty status.

One commonly suggested potential adverse consequence of case-based payment is that facilities may under-provide necessary services to control costs [17–19]. On the contrary, the increased number of outpatient visits observed in this study and qualitative findings from the study suggest that patients were in fact better managed, i.e. more in line with established clinical guidelines. This was probably mainly because the payment standard for the outpatient package was generous in terms of covering the full cost of services and drugs recommended by the clinical treatment guideline.

While the reduced gap in terms of inpatient service use between poor and non-poor TB patients indicates improved equity, it remains difficult to assess with existing data if the high levels of hospitalization following the intervention implementation represent an improvement in service provision, as a majority of TB patients may only require outpatient treatment according to global and national treatment guidelines. The original case-based payment design was a standard payment rate for inpatient and outpatient TB services combined, aiming to promote substitution of hospitalization by outpatient treatments and to avoid unnecessary hospitalization. However, during the program implementation, significant challenges were encountered from hospitals and health insurance agencies in program regions due to expected reduction in revenue, and difficulty in management. As a result, two payment packages were implemented for inpatient and outpatient services separately, the design of which was unable to reduce hospitalization. The detailed implementation challenges and consequences were discussed in detail in another paper by the same study team. Nevertheless, the decreasing gap in inpatient service utilization between the extreme-poverty group and non-poverty group could have positive effects, as qualitative interviews revealed that many extreme-poverty patients were elderly patients and had several co-morbidities (such as diabetes, hypertension, and kidney diseases) or side effects from TB treatment. Better access to inpatient services may improve the management of complex TB cases with co-morbidities. Future studies are needed to access the health service quality and appropriateness, and treatment outcomes.

The pro-poor effects can be explained by several features. First, the program required reimbursement rates for all insurance schemes to be no less than 70% for covered inpatient and outpatient services. Before the program, there was wide variation in reimbursement rates by the three major health insurance schemes. NCMS and URBMI reimbursed 60–75% of inpatient expenses, whereas UEBMI reimbursed 85–95% [11]. NCMS and URBMI had no or low reimbursement rates for outpatient expenses [11]. Poor TB patients usually enrol in NCMS and URBMI schemes which have lower reimbursement rates. The program reduced the disparity in reimbursement rates, thereby favouring the poor. Second, an upgraded IT system that allowed immediate insurance reimbursement was most beneficial for poorer patients, as they were not required to pay a large amount of cash at the point of service use, and then got a reimbursement later. Third, transport and subsistence allowances provided strong incentives to poor patients, many of whom live in remote rural areas and had higher transportation costs, to seek care. Fourth, medical financial assistance was provided by the Department of Civil Affairs for a relatively small number of eligible low-income households, further reducing their financial burden. Fifth, from the supply side, the implementation of case-based payment, clinical pathways, and case management probably improved the treatment and management quality more in under-developed areas, where many poor patients resided.

Despite great improvements in TB management, poor TB patients still face a number of barriers and challenges in accessing health care and completing treatment. Some TB services and drugs were excluded from insurance program reimbursement. For example, in many counties, CT scans, branded liver-protection drugs and ancillary drugs were not covered by insurance, nor restricted by the cost limit of case-based payment [6]. Though case-based payment was designed to restrict the provision of unnecessary tests and drugs, their actual use was not uncommon. For instance, a significant proportion of patients reported having adverse reactions to free anti-TB drugs, and were therefore given more expensive second line drugs and in some cases were encouraged to take liver-protection drugs, or be admitted for inpatient care. In order to further reduce the financial barriers to care for poor TB patients, the types of services covered by insurance should be expanded, with appropriate safeguards to control unnecessary use of more expensive treatments.

The study was not without limitations. There was very limited quantitative information on patient case-mix and service details (such as prescriptions and procedures). Therefore, appropriateness and quality of services cannot be assessed objectively. Service quality can only be inferred from reported patient satisfaction and qualitative feedback from patients, doctors and administrators. Besides, the program consists of multiple concurrent interventions that may be synergistic or antagonistic, and thus the effects of each individual intervention cannot be evaluated separately.

## Conclusions

The China-Gates TB program Phase II effectively improved TB patients’ access to inpatient and outpatient services and improved medication adherence across all income groups. Patients in households classified as in extreme or moderate poverty benefited more from the inpatient service coverage of the program compared to a non-poverty group, indicating improved equity in inpatient TB service access. The pro-poor design of the program provides important lessons to other TB programs in China and other countries to better address TB care for the poor. The study also identified other difficulties poor TB patients face that need to be further addressed.

## Additional file

Additional file 1: Multilingual abstracts in the five official working languages of the United Nations. (PDF 468 kb)

## Data Availability

The datasets generated and analysed during the current study are not publicly available due to the regulations of China CDC. Readers of the article need to discuss with China CDC and obtain their permission before the release of the dataset.

## Abbreviations

China CDC: Chinese Center for Disease Control and Prevention
CT: Computed tomography
FGD: Focus-group discussion
NCMS: New cooperative medical schemes
TB: Tuberculosis
TBIMS: Tuberculosis information management system
UEBMI: Urban employee basic medical insurance
URBMI: Urban resident basic medical insurance
WHO: World Health Organization
