# Supplementary material for: Impact of an innovative tuberculosis financing and payment model on health service utilization by tuberculosis patients in China: do the poor fare better than the rich?
Source: Infect Dis Poverty. 2019 Jun 11;8:44. doi: 10.1186/s40249-019-0559-z (PMC6558905; doi:10.1186/s40249-019-0559-z)
Supplement: Supplementary file 1 — Multilingual abstracts in the five official working languages of the United Nations. (PDF 468 kb) [file 40249_2019_559_MOESM1_ESM.pdf]

Translation of the abstract into the five official working languages of the United Nations

تأثير نموذج مبتكر للتمويل وللدفع للخدمات الصحية التي ينتفع بها مرضى السل في الصين: هل الفقراء يصبحون أفضل من الأغنياء؟

دي دونج، ووي شيانغ جيانغ، وتشيان لونغ، وفيي هوانغ، هوى تشانغ، وجيا ينغ تشن، ولي شيانغ، وتشيانغ لي، وشنغ لان تانغ، وهنري لوكاس

نبذة مختصرة
معلومات أساسية: يرتبط انتشار السل (TB) ارتباطًا وثيقًا بالفقر في الصين ، ويواجه المرضى الفقراء المزيد من العوائق أمام العلاج. باستخدام نهج قائم على التأمين، تم تنفيذ المرحلة الثانية من برنامج تشاينا جيتس للسل "China-Gates TB" بين عامي 2012 و2014 في ثلاث مدن في الصين لتحسين الوصول إلى الرعاية ضد مرض السل وتخفيف العبء المالي على المرضى، وخاصة بين الفقراء. تهدف هذه الدراسة إلى تقييم آثار البرنامج على استخدام الخدمة وإنعكاسها المنصف على مجموعات الدخل المختلفة.
الأساليب: كانت بيانات 788 و775 مريض في التقييم الأساسى والنهائي متاحة للتحليل بالتتابع. تم تقييم استخدام خدمة المرضى المقيمين وبالعيادات الخارجية، والالتزام بالعلاج، ورضا المرضى قبل البرنامج وبعده، وعبر فئات الدخل المختلفة (الفقر المدقع ، الفقر المعتدل وعدم الفقر)، وفي مدن البرامج المختلفة، باستخدام الإحصاءات الوصفية ونماذج التراجع متعدد الأشكال. وأجريت مقابلات مع أصحاب المصلحة الرئيسيين لتقديم تقييم بخصوص تطبيق البرنامج ونتائجه.
النتائج: بعد تطبيق البرنامج، زاد معدل دخول مجموعة الفقر المدقع للمستشفى بنسبة أكثر (48.5 ٪ إلى 70.7 ٪) ومجموعة الفقر المعتدل (45.0 ٪ إلى 68.1 ٪)، مقارنة مع المجموعة الغير فقيرة (52.9 ٪ إلى 64.3 ٪). وكانت أكبر زيادة أيضا في عدد زيارات مجموعة الفقر المدقع للعيادات الخارجية (4.6 إلى 5.7). زادت نسبة المرضى الذين إلتزموا بشكل جيد بالدواء بنسبة 15 نقطة مئوية في مجموعة الفقر المدقع وعشر نقاط مئوية في المجموعات الأخرى. كانت معدلات الرضا عالية في جميع المجموعات. كما اقترح أصحاب المصلحة في تقييمهم لنوعية الخدمة، بأن زيادة معدلات التعويضات، وتسهيل إجراءاتها، والبدل، قد أدى إلى تحسن استخدام الخدمة للمرضى. إن تطبيق الدفع القائم على الحالات قد جعل توفير الخدمات أكثر توافقًا مع المسارات السريرية.
الاستنتاجات: استفاد المرضى الذين يعيشون في فقر مدقع أو معتدل أكثر من البرنامج مقارنةً بمجموعة غير فقيرة، مما يشير إلى تحسن العدالة في الوصول إلى خدمة علاج السل. يوفر تصميم البرنامج المؤيد للفقراء دروسًا هامة لبرامج السل الأخرى في الصين والبلدان أخرى، عن الاهتمام برعاية فقراء مرضى السل بشكل أفضل.

Translated from English version into Arabic by Mai Ghanem, Revised by Ghada Abaza, through

TRANSLATORS
WITHOUT BORDERS

创新型筹资支付模式对中国结核病患者卫生服务利用的影响：是否贫困患者更多受益？

Di Dong, Wei-Xi Jiang, Qian Long, Fei Huang, Hui Zhang, Jia-Ying Chen, Li Xiang, Qiang Li, Sheng-Lan Tang and Henry Lucas

摘要

引言：中国结核病的患病率与贫困有明显的相关性。贫困患者治疗面临更多的困难。通过医疗保险手段，中国-盖茨基金会结核病项目二期于 2012 年至 2014 年间在中国三个地市推广，目的为提高结核病医疗服务的可及性并减轻患者的经济负担，尤其是针对贫困患者。本研究的目的为评估项目对服务利用的影响，以及对不同收入组患者的影响和公平性。

方法：数据分析使用了基线调查的 788 名患者信息和终末调查的 775 名患者信息。研究利用描述性统计分析和多变量回归模型分析了项目前后、不同收入组（极端贫困，中度贫困，和

非贫困）、和不同地区间结核病患者的住院和门诊服务利用、治疗依从性和患者满意度指标。也进行了关键人员访谈从而定性评估项目的执行和效果。
结果：项目推广后，住院率在极端贫困组（48.5%到 70.7%）和中度贫困组（45.0% 到 68.1%）中提高多于非贫困组（52.9% 到 64.3%）。门诊服务利用次数也在 极端贫困组中提升最多（4.6 次到 5.7 次）。治疗服药依从性较好的患者比例在极端贫困组中提升了 15 个百分点，在其他收入组提升了 10 个百分点。满意度在所有收入组中均有提高。关键人物访谈也发现更高的报销比例、更容易的报销手续和补贴提高了患者的服务利用。按病种付费的推广也促进了更符合临床路径的诊疗。
结论：极端贫困和中度贫困的患者从本项目中的受益多于非贫困患者，说明项目促进了结核病服务的公平性。中盖项目向贫困患者倾斜的项目设计为中国和其他国家更好的促进贫困结核病患者的治疗提供了重要的经验。

Translated from English version into Chinese by Di Dong

Impact d'un modèle innovant de financement et de rémunération sur l'utilisation des services de santé par les patients tuberculeux en Chine : le traitement est-il plus abordable pour les plus démunis ?

Di Dong, Wei-Xi Jiang, Qian Long, Fei Huang, **Hui Zhang**, Jia-Ying Chen, Li Xiang, Qiang Li, Sheng-Lan Tang et Henry Lucas

Résumé

Contexte : La prévalence de la tuberculose est étroitement associée à la pauvreté en Chine et ce sont les patients pauvres qui rencontrent le plus d'obstacles pour se faire soigner. La Phase II du programme China-Gates TB, suivant une approche basée sur l'assurance, a été exécutée entre 2012 et 2014 dans trois villes de Chine dans le but d'améliorer l'accès au traitement de la tuberculose et de réduire le fardeau financier pour les patients, notamment les plus démunis. La présente étude évalue les effets du programme sur l'utilisation des services et sur l'équité entre les différents groupes de revenus.
Méthodes : les données de 788 patients au début de l'étude et 775 lors de l'évaluation finale étaient disponibles pour l'analyse. L'utilisation des services d'hospitalisation et de traitement ambulatoire, l'observance du traitement et la satisfaction des patients ont été évaluées avant et après le programme dans différents groupes de revenus (pauvreté extrême, pauvreté modérée, pas de pauvreté) et différentes villes du programme, à l'aide de statistiques descriptives et de modèles de régression multivariée. Des entretiens ont été menés avec les parties prenantes importantes afin d'évaluer l'exécution et l'impact du programme du point de vue qualitatif.
Résultats : Après la mise en application du programme, le taux d'hospitalisation a davantage augmenté pour les groupes de pauvreté extrême (de 48,5 % à 70,7 %) et de pauvreté modérée (de 45,0 % à 68,1 %) que dans le groupe de non-pauvreté (de 52,9 % à 64,3 %). La plus grande augmentation du nombre de consultations externes s'observait également dans le groupe de pauvreté extrême (de 4,6 à 5,7). La proportion de patients qui respectaient bien leur traitement médicamenteux a augmenté de 15 points dans le groupe de pauvreté extrême et 10 points dans les autres groupes. Les taux de satisfaction étaient élevés dans tous les groupes. Le retour d'information qualitatif des parties prenantes suggérait également qu'une augmentation des taux de remboursement, des démarches de remboursement plus faciles et une indemnité amélioraient l'utilisation des services par les patients. La mise en place d'une rémunération au cas a ramené la prestation de services dans la ligne des parcours cliniques.

Conclusions : Les patients vivant dans une pauvreté extrême ou modérée ont tiré davantage de bénéfice du programme que ceux qui n'étaient pas pauvres, ce qui indique une amélioration de l'équité dans l'accès aux services de traitement de la tuberculose. On peut tirer de la conception favorable aux plus pauvres du programme des enseignements importants pour d'autres programmes de lutte contre la tuberculose en Chine et dans d'autres pays, dans le but d'améliorer le traitement des plus démunis.

Translated from English version into French by Suzanne Assenat, Revised by Charlotte Thomas, through

Влияние инновационной системы финансирования и оплаты медико-санитарных услуг для больных туберкулезом в Китае: делает ли это стоимость услуг для малоимущих лучше, чем для обеспеченных?

Ди Донг, Вей-Си Цзян, Цянь Лун, Фей Хуан, **Хуи Чжанг**, Цзя-Ин Чен, Ли Сян, Цян Ли, Сэн-Лан Танг и Генри Лукас

Аннотация

Предпосылки: Распространение туберкулеза (ТБ) в Китае тесно связано с бедностью, при этом малообеспеченные больные сталкиваются с большим количеством проблем при лечении. Используя подход, основанный на страховании, программа по борьбе с туберкулезом China-Gates TB фаза II проводилась с 2012 по 2014 годы в трех городах Китая для расширения доступа к лечению туберкулеза и уменьшения финансового бремени на пациентов, особенно среди малообеспеченного населения. Целью настоящего исследования является оценка эффективности действия программы в отношении использования услуг, а также влияния программы на обеспечение равенства различных групп населения с разным доходом.

Методы: анализ был проведен на основании данных 778 и 775 пациентов по исходным показателям и по окончательной оценке, соответственно. Использование услуг амбулаторной и стационарной помощи, соблюдение требований лечения, а также удовлетворенность больного оценивались до и после проведения программы в группах населения с разным уровнем дохода (крайняя бедность, умеренная бедность, группы, не относящиеся к малообеспеченным) и в различных городах, участвующих в программе, с использованием описательной статистики и моделей множественной регрессии. Для количественной оценки выполнения программы и ее влияния опрашивались ее ключевые участники.

Результаты: после реализации программы количество принятых больных в больницах увеличилось в большей степени в отношении группы населения, живущей в условиях крайней бедности (с 48,5% до 70,7%), и в умеренно бедной группе (с 45,0% до 68,1%) по сравнению с группой, не относящейся к малообеспеченным (с 52,9% до 64,3%). Наибольший рост числа амбулаторных посещений был также в группе, живущей в условиях крайней бедности (с 4,6 до 5,7). Доля больных с хорошим уровнем соблюдения процедуры лечения увеличилась на 15 процентов в группе, живущей в условиях крайней бедности и на 10 процентов в других группах. Степень удовлетворенности была высока во всех группах.

Количественная обратная связь от участников указывает на то, что увеличение сумм возмещения, упрощение процедуры получения возмещения, а также доплаты улучшили пользование услугами со стороны больных. Применение оплаты в зависимости от конкретной ситуации привело к лучшему соблюдению клинических протоколов при оказании услуг.
Заключение: больные из групп, живущих в условиях крайней или умеренной бедности, получили больше преимуществ от программы по сравнению группой, не относящейся к малообеспеченной, что указывает на улучшение соблюдения принципа равенства в доступе к противотуберкулезному лечению. Благодаря направленности программы на бедные слои населения другие программы по борьбе с туберкулезом в Китае и других странах получили важные данные для более эффективного лечения туберкулеза среди бедных слоев населения.

Translated from English version into Russian by Irina Nosova, Revised by Alexander Somin, through

TRANSLATORS
WITHOUT BORDERS

El impacto de un modelo innovador para la financiación y el pago de los gastos relacionados con la tuberculosis sobre la utilización de servicios sanitarios por parte de los pacientes con tuberculosis en China: ¿les va mejor a los pobres que a los ricos?

Di Dong, Wei-Xi Jiang, Qian Long, Fei Huang, **Hui Zhang**, Jia-Ying Chen, Li Xiang, Qiang Li, Sheng-Lan Tang y Henry Lucas

Resumen

Antecedentes: en China, la prevalencia de la tuberculosis (TB) está estrechamente relacionada con la pobreza, y los pacientes pobres se enfrentan a más barreras para el tratamiento. Utilizando un enfoque basado en seguros, la Fase II del programa China-Gates para el control de la TB se implementó entre 2012 y 2014 en tres ciudades chinas para mejorar el acceso a la atención de la TB y reducir la carga financiera para los pacientes, especialmente los pobres. El presente estudio pretende evaluar los efectos del programa sobre el uso de los servicios, así como su impacto sobre la equidad entre distintos grupos de ingresos.
Métodos: para el análisis estuvieron disponibles los datos de 788 y 775 pacientes al inicio y la evaluación final, respectivamente. La utilización de los servicios hospitalarios y ambulatorios, la adherencia al tratamiento y la satisfacción de los pacientes se evaluaron antes y después del programa, en distintos grupos de ingresos (pobreza extrema, pobreza moderada y fuera de la pobreza) y en varias ciudades del programa, utilizando estadística descriptiva y modelos de regresión multivariantes. Se llevaron a cabo entrevistas con las partes interesadas más importantes a fin de evaluar de manera cualitativa la implementación y los efectos del programa.
Resultados: tras la implementación del programa, la tasa de hospitalización aumentó más para el grupo de pobreza extrema (48,5 % al 70,7 %) y el grupo de pobreza moderada (45,0 % a 68,1 %), en comparación con el grupo fuera de la pobreza (52,9 % a 64,3 %). El mayor incremento del número de visitas ambulatorias también fue para el grupo de pobreza extrema (4,6 a 5,7). La cantidad de pacientes con buena adherencia a los medicamentos aumentó en 15 puntos porcentuales en el grupo de pobreza extrema y en 10 puntos porcentuales en los demás grupos. Los índices de satisfacción fueron altos en todos los grupos. Asimismo, las observaciones cualitativas de las partes

interesadas sugirieron que las mayores tasas y más sencillos procedimientos de reembolso, así como la subvención mejoraron la utilización de los servicios por parte de los pacientes. La implementación del pago según cada caso hizo que la prestación de servicios fuera más compatible con las vías clínicas.
Conclusiones: los pacientes en pobreza extrema o moderada se beneficiaron más del programa en comparación con el grupo fuera de la pobreza, lo que indica una mayor equidad en el acceso a los servicios de TB. El diseño del programa a favor de los pobres aporta lecciones importantes para otros programas de TB en China y otros países para abordar mejor la atención de la TB para los pobres.

Translated from English version into Spanish by Mayra León, Revised by Lindsey Hoemann, through

TRANSLATORS
WITHOUT BORDERS
